# What Motivates Parents of Young Children With Down Syndrome to Participate in Research: A Focus Group Analysis

**DOI:** 10.1111/jar.70178

**Published:** 2026-01-12

**Authors:** Benedetta Ceci, Madison M. Walsh, Sara Colaianni, Miranda E. Pinks, Sara Onnivello, Kaylyn Van Deusen, Francesca Pulina, Chiara Marcolin, Bethany A. Gray, Elisa Rossi, Erika Lupati, Margherita Pietrobon, Alessandra Tomè, Deborah J. Fidler, Silvia Lanfranchi

**Affiliations:** ^1^ Department of Developmental Psychology and Socialization University of Padova Padua Italy; ^2^ Department of Human Development & Family Studies Colorado State University Fort Collins Colorado USA; ^3^ Department Clinical Psychology Colorado State University Fort Collins Colorado USA

**Keywords:** community based participatory research (CBPR), down syndrome (DS), focus groups, intellectual disability (ID), parents

## Abstract

**Background:**

This cross‐national study aimed to investigate and identify the motivations that drive parents of children with Down syndrome (DS) towards or away from participating in research.

**Methods:**

Participants were 33 parents of children with DS who took part in nine focus groups in the United States and in Italy. Answers to questions regarding motivation for research participation were transcribed and then analyzed using ATLAS.ti.

**Results:**

Six themes emerged regarding parent motivation: (a) Increasing parent knowledge; (b) Interest in a specific research topic; (c) Opportunities to receive support; (d) Logistical accessibility; (e) To support the DS community; and (f) To create a more inclusive society. ‘Interest in a specific research topic’ and ‘Logistical accessibility’ emerged as the main motivating factors cross‐culturally. Only subtle differences emerged between the two countries.

**Conclusion:**

Results of this work can inform the alignment of future DS research with community and stakeholder priorities and values.

## Summary

- The study explores parental motivations to participate in research amongst families with a child with Down syndrome in Italy and the US.
- Cross‐culturally, ‘Interest in a specific research topic’ and ‘Logistical accessibility’ emerged as the main motivating factors.
- The findings support more inclusive, community‐engaged research designs and help align scientific goals with the real needs of families and individuals with Down syndrome.

## 1 Introduction

In 2021, the United States National Institutes of Health (2021) highlighted the need for community‐engaged research to advance science and practise for individuals with Down syndrome (DS), the most common chromosomal cause of intellectual disability (ID) (Antonarakis et al. 2020). This call for greater community engagement emphasises the importance of collaborating with members of the DS community throughout the research process and aligns with broader efforts in health and behavioural science to implement community‐based participatory (CBPR) principles. CBPR is a research framework wherein researchers and community members (e.g., self‐advocates, caregivers, family members, and practitioners) become partners in the research process (Israel et al. 2010). This framework transforms a typical ‘top‐down’ research approach in which the academic community sets the agenda regarding the study of a population and instead encourages researchers to align their inquiry with the needs expressed by community members, involving them in the research process and working collaboratively (Wallerstein and Duran 2010). In research with individuals with DS, the co‐creation of knowledge between researchers, care providers, interventionists, educators, and family members can promote well‐being and quality of life (Fidler et al. 2003).

### 1.1 CBPR Principles

A CBPR framework involves the application of specific principles and practises in the research process (Israel et al. 1998, 2010). The present study focuses on two of these principles as a critical foundation for community‐engaged DS research. The first principle is that researchers and community members should act as collaborators throughout the research process (Riggs et al. 2020). Utilising this principle in the design of new research endeavours makes it so that community member priorities and topics of interest inform scientific inquiry. Members from within the DS community are viewed as collaborators who provide valuable lived experiences and expertise. Consequently, the interaction between community stakeholders and researchers can provide unique perspectives regarding research studies. By involving community members in the research enterprise, researchers can gain insights regarding lived experiences and can align research activities with priorities that are relevant to the community.

The second CBPR principle that informs the present study emphasises research as a cyclical and iterative process (Riggs et al. 2020). In order to foster a productive connection with the community, researchers must develop, maintain, and enhance partnerships with community members through ongoing processes. This cyclical and iterative relationship between researchers and community members may involve gathering formative data, defining issues and opportunities, developing research methodology, interpreting data, determining action plans and policy implications, and disseminating findings. This process involves ongoing dialogue with community members to hear ideas, perspectives, and priorities for advancing the well‐being of their community.

### 1.2 Understanding Motivation to Participate in Research

As an initial step towards building strong partnerships with DS community members, researchers must develop a genuine understanding of their contemporary priorities and preferences. This involves taking time to identify the underlying motivations that lead community members to volunteer for research participation and what benefit they are seeking from the experience. Amirav et al. (2017) highlighted the importance of motivation in determining parental participation and engagement and provided an explanation about the necessity for parents to participate in research that may be relevant to them and their children. Developing this awareness can enable researchers to approach the research partnership process from a place of respect for their community of interest, which can lead to a strong reciprocal relationship over time.

Parental motivations to participate in DS research have received increasing attention in recent years. A number of studies have addressed this topic across different types of DS research, from genomic data sharing to clinical and early‐life studies, and several common themes emerge that make parents of a child with DS more likely to enrol in a study, despite differences in study context. Burstein et al. (2014) and Menon et al. (2025) investigated parents' propensity to share genomic and open‐access data, finding that their motivation lies in promoting high‐quality research, helping generate more reliable data, and embodying altruistic values such as the desire to help others. At the same time, parents expressed concerns about data breaches, misuse, and privacy violations, as well as fears that results could have negative consequences for the DS population. All of these factors influence their willingness to participate and represent specific issues when discussing open‐access data.

Similar patterns of motivation, particularly those rooted in altruistic intentions, along with concerns about long‐term safety, are also evident in research involving medical interventions. Reines et al. (2017) focused their work on participation in pharmacological clinical trials, finding that parents were motivated by the potential benefits such interventions could bring to their child and to the DS community. However, concerns about long‐term safety, possible risks, and adverse drug effects limited their enthusiasm for participation, as did practical factors such as proximity to clinical trial sites and the impact of the study schedule on everyday life.

In addition to studies on medical and pharmacological interventions, parental motivations have also been examined in research involving families of newborns, where different practical and emotional factors come into play. Williams et al. (2018) explored factors that facilitate or hinder the recruitment of families with a newborn with DS from professionals' perspectives. Paediatricians, nurses, and support workers reported that families who agree to participate in research are motivated by a desire to support other parents, and that the sense of assistance and encouragement they may receive during challenging times can encourage them to enrol in birth cohort studies, as can higher educational background and socioeconomic status. Specific to this kind of research is the importance of factors such as the child's health status at birth and any required surgery; the quality of communication when delivering the DS diagnosis and the modalities of family recruitment, which can affect future relationships with medical professionals and researchers; the time commitment and the design of the study to fit into life with a newborn; and the differences in the medical care pathways for children with DS across regions.

Taken together, these studies highlight how parental motivations vary across different research contexts. Building on this broader perspective, White et al. (2022) investigated parents' motivations to participate in a range of DS research studies, including medical, pharmacological, observational, and survey‐based studies. The authors found that parents were willing to contribute to scientific studies primarily because of the potential benefits for their own child and for the broader DS community. However, this motivation was closely associated with the need for transparency regarding the study's aim, access to results—especially medical ones—and assurance that people with DS would genuinely benefit from the research. Additional factors influencing willingness to participate included the child's perceived cooperation and engagement, the parent's availability and competing responsibilities, ethical and privacy considerations, and study inclusion or exclusion criteria. Parents also identified several barriers to participation, such as concerns about potential risks and child well‐being, the adequacy of study compensation, and the presence of invasive procedures (e.g., completing surveys or interviews versus providing blood samples, taking medication, or receiving injections). Furthermore, logistical challenges such as travel distance and time commitment were amongst the most frequently reported barriers. As Bardhan et al. (2024) note, reducing such practical barriers may encourage participation and strengthen collaborative, community‐based approaches to research.

Collectively, these studies highlight both the personal and social nature of parental motivations, sharing some themes about DS research participation of families, but also providing specific parental motivations according to the particular context in which the study is conducted. In fact, most existing work remains focused on medical or pharmacological contexts, predominantly in the United States (e.g., Burstein et al. 2014; Bardhan et al. 2024; Reines et al. 2017), whilst evidence from the psychological domain is still limited. The present study addresses this gap by expanding the limited body of evidence on parents' motivations to participate in psychological studies and adopting a cross‐national approach.

Exploring this topic across countries is particularly valuable, as parental motivations and attitudes towards research may be shaped by cultural values and the structure of healthcare systems. Cross‐national comparisons make it possible to distinguish culture‐specific influences from shared motivational patterns, enabling researchers to identify overarching themes and develop comparable goals and procedures that can be applied across contexts. This, in turn, promotes greater methodological coherence, enhances international collaboration, and supports the development of globally relevant ethical and research practises that foster more inclusive participation.

To explore these questions, a focus group approach was employed in the present study, as it allows participants to discuss their perceptions, ideas, and opinions (Krueger and Casey 2000). Applied to community engaged research in DS, this method helps researchers better understand their co‐partners in the research process and ultimately align their investigations with the priorities expressed within the DS community.

The goal of this work was to identify the factors that increase and decrease motivation for research participation amongst families of young children with DS. Parents from both Italy and the US participated in focus groups and responded to questions regarding what makes them interested in participating in research and what limitations they perceive when deciding whether to take part in a study. Their responses were then transcribed and analysed using a qualitative thematic approach. Cross‐national comparisons were also examined. The findings contribute to a broader shift in research on neurogenetic conditions associated with ID and better align new research directions with the preferences of those who are members of the community.

## 2 Materials and Methods

### 2.1 Participants

Participants were 34 parents of young children with DS. Participants were between 29 and 54 years old (*M* = 42.06, SD = 5.92), and most identified as females (94.18%). Participants reported advanced levels of education. One participant did not provide demographic information.

Sociodemographic information is presented in Table 1.

**TABLE 1:** Summary of the socio‐demographic characteristics of participants.

	Italian participants	American participants
*N*	19	14
Age *M* (SD)	45.22 (4.71)	37.72 (5.16)
Education % (*n*)		
Master's degree	68.4% (13)	31.3% (5)
Bachelor's degree	5.3% (1)	37.5% (6)
Associate degree	0	12.5% (2)
High school diploma	22.1% (4)	6.3% (1)
Not reported	5.3% (1)	22.1% (4)
Sex % (*n*)		
Female	89.5% (17)	100% (14)
Employment % (*n*)		
Full‐time employment	57.9% (11)	18.8% (3)
Part‐time employment	26.3% (5)	37.5% (6)
No employment	10.5% (2)	25% (4)
Not reported	5.3% (1)	10.5% (2)

### 2.2 Procedure

Participants were recruited in the United States and Italy through flyers, either sent by email to families already known to the research team or distributed through local and regional DS advocacy organisations and medical centres. Most participants were parents of preschool‐aged children with DS and one participant was a parent of a school‐age child with DS. Nineteen participants were Italian, and 14 were American. Informed consent was completed before participants started the project. This study was carried out in accordance with the protocol approved by the Ethics Committee of the School of Psychology at the University of Padua ethics committee and the Institutional Review Board of Colorado State University Institutional Review Board.

### 2.3 Focus Group Interviews

Focus groups were held virtually due to COVID‐19 safety precautions in place at the time of the study. Nine virtual focus groups were conducted; 6 were conducted in the US and 3 were conducted in Italy. Each focus group was led by two facilitators and lasted approximately 1–2 h. Focus groups were conducted in English in the US and in Italian in Italy.

Focus group facilitators were trained to maintain a positive and engaged attitude throughout the focus group discussion to help participants feel at ease and to promote conversation amongst the participants. Discussion questions focused on general topics related to research participation, parents' developmental goals and priorities for their children. The present study analysed responses to the first two questions posed in each discussion, both relating to motivation for research participation: (1) ‘What makes you interested in participating in research?’ and (2) ‘What makes you less interested in participating in research?’. Parent responses to the other discussion questions are presented elsewhere (Walsh et al. 2024).

All focus groups were conducted and recorded using a secure video conferencing platform (Zoom in Italy and Microsoft Teams in the US). Recordings from the Italian focus groups were transcribed by a research associate and reviewed for accuracy by the discussion facilitator. Recordings from the US focus groups were transcribed automatically by the Microsoft Teams platform and reviewed independently by two research associates. In both countries participant names and other personally identifiable information mentioned by parents (e.g., child names, school names, sibling names) were removed from the transcripts before analysis. Italian transcripts were translated into English for analysis. Italian transcripts were translated by two bilingual (Italian and English) members of the Italian research team. Translated transcripts were then reviewed for grammar by an English‐speaking American research team member involved in other unrelated research projects. This American team member was naïve to the study objectives and did not attend project meetings, and, therefore, was understood to be an unbiased translation reviewer. Before analysis, facilitator comments were removed from the transcripts, leaving only the participant responses.

### 2.4 Data Analysis Plan

#### 2.4.1 Data Coding

Coding the transcripts involved manual and automatic processes. First, transcripts were reviewed by the first author, and six recurring themes were identified: (1) Increasing parent knowledge; (2) Interest in specific research topics; (3) Opportunities to receive support; (4) Logistical accessibility; (5) Supporting the DS community; and (6) Creating a more inclusive society. After these themes were identified, a list of keywords related to each theme was developed collaboratively by the two cross‐national research teams. This iterative development process aimed to reduce cultural biases in the language used. After the list of keywords was developed, transcripts were coded automatically using ATLAS.ti Web (version 5.8.0). Using the electronic searching feature in ATLAS.ti 2021, parent responses that mentioned a word on the keyword list were automatically coded. Then, coded quotations were reviewed by the first author and determined to be either ‘on‐topic’ or ‘off‐topic.’ Coded parent responses were determined to be ‘on‐topic’ if the quote included a word in the keyword list and was related to an associated theme. Parent responses were determined to be ‘off‐topic’ if the quote included a word in the keyword list, but the word was used in a different context or with a different meaning and therefore the quote did not actually relate to one of the six themes. This was an important step because some parent responses included keywords that were not related to the associated theme and, therefore, were not coded. Additionally, some keywords were used for more than one theme. Therefore, the first author also reviewed the automatically coded responses and chose the appropriate theme based on the theme definition.

Reflexivity was incorporated throughout the analytic process. The research team engaged in joint discussions when developing interview questions and interpreting participants' responses and themes, whilst small groups working with the data conducted in‐depth reflections to ensure that multiple perspectives were taken into account. Further details are provided in (Walsh et al. 2024).

Themes and keyword lists are presented in Table 2.

**TABLE 2:** Themes and associated keywords.

Themes	Definition	Keyword list
Increasing parent knowledge	The parents are linked to the possibility of obtaining new information or the necessity related to acquiring and comprehending more information about the field of research.	Research; Knowledge; Opportunities; Useful; Information; Study; Direct the behaviour; Found; Understand; Podcast and webinar; Finding; Discovery; Insight
Interest in a specific research topic	The parent indicates an interest in participating in research or, more broadly, when the research objective or project contains themes in which they are interested.	Interest; Topic; Child with special needs; Down syndrome; My child/son/daughter; Involve me; Area of development; Closer to him/her; Our life; Needs; Benefit; Disabilities; Skill; Goal
Opportunities to receive support	The parents mentioned the advantages of getting guidance from a specialist, not only in person but even online.	Someone else; Objective look; Comparison; Guide; Expert; Podcast and webinar; Guideline; Early intervention (system of support/expert that helps you); Follow; Help; Provide; Service; Psychologist; Therapist; Doctor; Educator; Specialist
Logistical accessibility	The parent talks about the time and energy required to participate in a research study, whether positively or negatively.	Availability; Close (home); Commitment; Distance; Time (minutes/h); Schedule; Busy; Alone; Away; Home; City; Available; Flexibility; Manage
To support the DS Community	The parents use them to communicate the intention that by taking part in a research project, they will help collect data that will be beneficial to other families.	Gather data; Help; Contribution; Sharing; Useful; Progression; Duty; For Parents; Other child; Other parents; Other family; Other kid; Support
To create a more inclusive society	The parents stated that the goal of his acts was to build a better society, for both current and future generations.	Society; Other people; Integrate; Integrating; General; Include; Inclusion; Inclusive; Community; Culture; Others; People coming up

#### 2.4.2 Data Analysis

Coded data were exported as frequency tables and analysed in Microsoft Excel. Data visualisations were created to assist with interpreting the results. Two sets of analyses were performed: comparison within focus groups and a comparison between Italian and American responses. To determine how frequently participants provided responses related to each theme, frequency percentages were calculated for each focus group. This was achieved by dividing the number of quotations associated with a specific theme in a given focus group by the total number of quotations made during that group's discussion. For example, in the first focus group, participants made a total of 168 quotations, 29 of which were related to the theme ‘Increasing parent knowledge.’ As a result, 17.26% of the discussion in the first focus group focused on this theme. Additionally, the mean frequency percentage across the nine focus groups was calculated for all analysed themes. Subsequently, the frequency percentage related to each theme was calculated separately for US and Italian participants by dividing the number of quotations connected to each theme by the total number of quotations made within each country's focus groups. A Chi‐Squared analysis was then conducted to compare frequency trends between the Italian and US focus groups.

## 3 Results

### 3.1 Thematic Analysis Within Focus Groups

**FIGURE 1:**
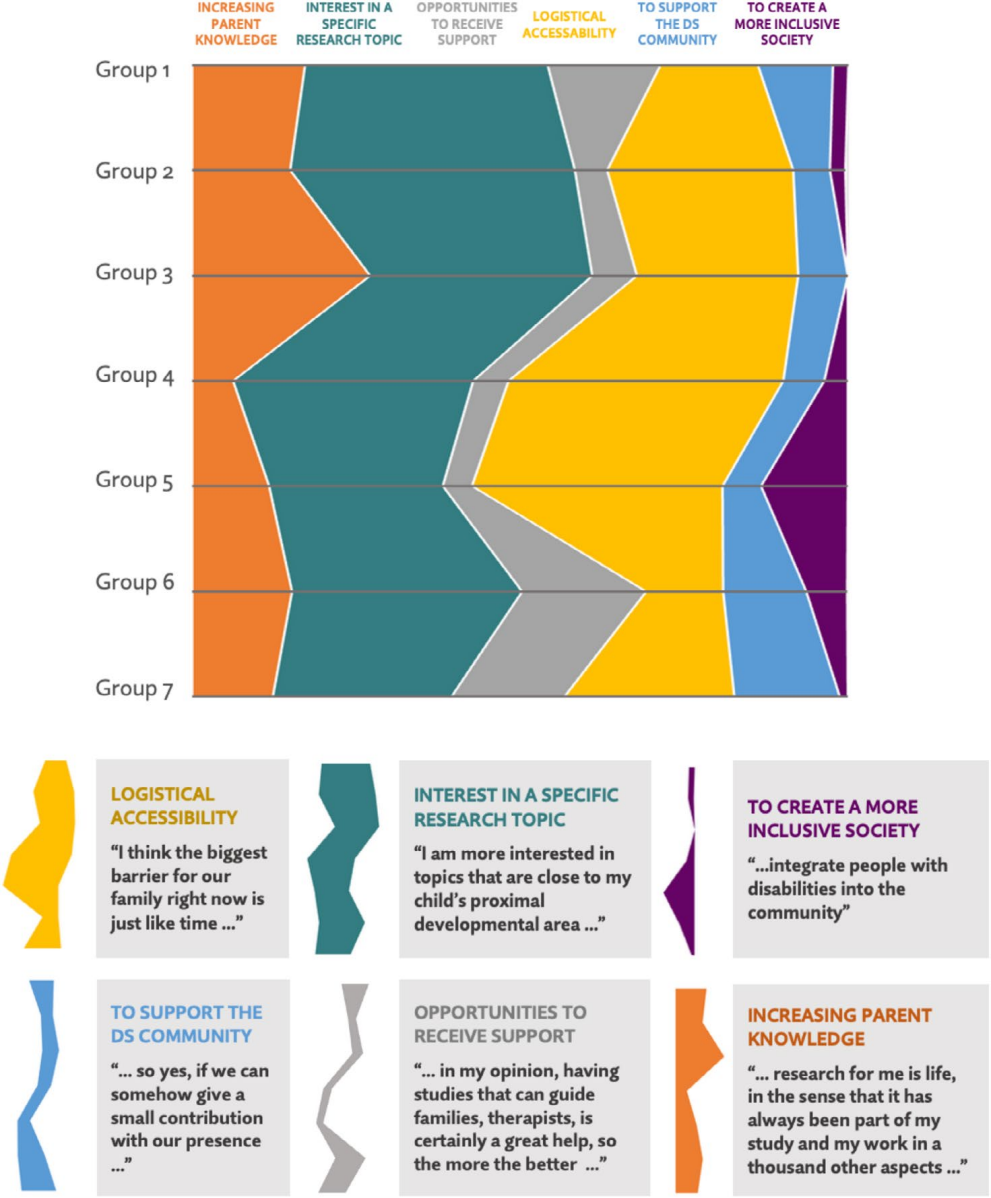
Themes frequencies for each focus group.

A total of 945 quotations from nine focus group transcripts were identified as relating to parental motivations to participate in research and were coded as pertaining to one of the six themes identified: (1) Increasing parent knowledge; (2) Interest in a specific research topic; (3) Opportunities to receive support; (4) Logistical accessibility; (5) To support the DS community; and (6) To create a more inclusive society. Codes by frequency for each focus group are reported in Table 3 and Figure 1. These percentages were calculated by dividing the number of citations for each theme by the total number of citations across all focus groups, reflecting the relative prominence of each theme in the discussions. The data represent the proportion of times participants emphasised these themes, highlighting their priorities and motivations for engaging in research.

**TABLE 3:** Percentage frequencies topic words for each focus group.

	FG 1	FG 2	FG 3	FG 4	FG 5	FG 6	FG 7	FG 8	FG 9	Total
Topic frequencies (%)	62	78	40	41	18	39	22	9	12	321
Interest in a specific research topic	36.90%	43.33%	33.90%	34.54%	26.47%	35.14%	27.16%	15.25%	29.27%	33.97%
Logistical accessibility	25	51	29	47	26	13	21	26	19	257
	14.88%	28.33%	24.58%	39.50%	38.24%	11.71%	25.93%	44.07%	46.34%	27.20%
Increasing parent Knowledge	29	27	32	7	8	17	10	10	5	145
	17.26%	15.00%	27.12%	5.88%	11.76%	15.32%	12.35%	16.95%	12.20%	15.34%
Opportunities to receive support	29	9	8	13	3	21	14	2	2	101
	17.26%	5.00%	6.78%	10.92%	4.41%	18.92%	17.28%	3.39%	4.88%	10.69%
To support the DS Community	19	10	9	7	4	14	13	8	2	86
	11.31%	5.56%	7.63%	5.88%	5.88%	12.61%	16.05%	13.56%	4.88%	9.10%
To create a more inclusive society	4	5	0	4	9	7	1	4	1	35
	2.38%	2.78%	0.00%	3.36%	13.24%	6.31%	1.23%	6.78%	2.44%	3.70%

Across all focus groups, the themes identified most frequently were ‘Interest in a specific research topic’ (33.97%) and ‘Logistical accessibility’ (27.20%). Other frequently mentioned themes were ‘Increasing their knowledge’ (15.34%), ‘Opportunities to receive support’ (10.69%), and ‘To support the DS community’ (9.10%). Lastly, ‘To create a more inclusive society’ was the least frequently mentioned theme (3.70%).

#### 3.1.1 Interest in a Specific Research Topic

‘Interest in a specific research topic’ was the most frequently mentioned theme across all focus groups. All participants referred to it at least once, accounting for 33.97% of the discussions. Participants reported higher motivation to engage in research when the project addressed themes of direct relevance to their and their children's lives: ‘I'm very grateful for all of the work that has been done that has helped benefit my daughter… So, I want to continue that in an area where there's limited resources and limited research that has been done’ and ‘…obviously Down Syndrome's near and dear to us because of our [child] and our [niece]’. Beyond general interest, parents emphasised more nuanced dimensions of relevance, such as the importance of research objectives being closely aligned with the challenges they face in their daily lives, in relation to their immediate applicability to their child's current stage of development: ‘…probably I would not be interested in participating in things that would not involve me from the point of view of age and area of development of the child […] I am more interested in topics that are close to my child's proximal development area’; ‘…the topics that can interest me are those closer to the age when [Child Name] is…’. Similarly, the perceived usefulness of the research objective and its potential to provide practical strategies were identified as key factors influencing parents' motivation to participate: ‘… we are all interested in research, also because it is our life, it is our world, so… everything I can know about Down syndrome is important to help [Child Name]’ and ‘…it is important because the more we talk about it, the more we deepen this topic, this situation, the more we can find strategies to give chances to these guys or to bring out the best’. A parent shared that developing a solid foundational understanding of the scientific aspects related to their child's challenges serves as a strong motivator for research participation: ‘I am a little bit interested in everything, for it is a source of personal baggage since [Child Name] was born, everything that can interest her’.

#### 3.1.2 Logistical Accessibility

The theme of ‘Logistical accessibility’ was discussed in all nine focus groups and mentioned by 32 out of 33 participants (18 Italians and all Americans), accounting for 27.20% of the discussions. Participants from the United States, in particular, highlighted the difficulty of reaching study locations due to long distances. Whilst some parents did not consider this a concern stating, for example, ‘…if there is someone able to do it from the other side of the world, it is a good thing and we take all the cues even if they are far away’ and ‘… the distance honestly doesn't worry me because it's a need of mine’, others identified it as a major barrier: ‘the distance is probably a huge limiting factor’ and ‘…location definitely does play a part as well…’. Time constraints were another frequent concern. Parents often reflected on the challenges of scheduling around daily routines and maintaining consistency with research requirements: ‘…it is always a question of timing, both in terms of time and frequency…’ and ‘Time is an issue for us… and I work part‐time and so trying to find the time for it is tricky’. Managing participation alongside work and school schedules appeared particularly challenging: ‘…set times of when it would run into the kids' school or my work…That would be a barrier’ and ‘…two jobs, a family, two children, it is always necessary to fit many things in, but for [Child Name] we do it, if it is not too much of a commitment, it can be done’. Indeed, the level of commitment was seen as a major factor. As one parent explained: ‘What makes the difference is the commitment that this implies, maybe in terms of moving, or going to attend courses, following therapy in person, schedules to be respected, this could put us in a bit of a crisis at a family logistic level’. Several parents also highlighted the emotional and physical toll involved: ‘…for her, it's stress away from home for many hours, the factor of getting dressed, leaving, the car, the very long journey… but it's also stressful for us, as parents, having to hold her all the time’. Despite these obstacles, many parents expressed greater willingness to participate when logistical demands were minimised. Motivation was closely tied to the perceived accessibility of the study: ‘…if once a year there is the need to travel to another city we have always done it […] But if it was a more daily thing clearly the distance becomes important’. As a result, flexibility was seen as essential: ‘Flexibility and time…is important’ and ‘… we do have jobs, so we would need it to be flexible with time’.

#### 3.1.3 Increasing Parent Knowledge

The theme ‘Increasing parent knowledge’ was discussed in all nine focus groups. Thirty‐one out of 33 participants (18 Italians and 13 Americans) mentioned ‘Increasing parent knowledge’ at least once, and it accounted for 15.34% of the discussions. Parents frequently linked their participation to the opportunity to gain new information and be updated on research: ‘all that can arrive, that… put us in relation with the knowledge for us is however important’ and ‘We would be interested even if it was not research but knowledge’. Another parent shared that ‘as a provider who works with lots of families, with kids with Down syndrome, it's nice to have like research, resources, and stuff I can give them’. At other times, parents noted that their participation in research isn't just about increasing their knowledge, but also about meeting the need to acquire and understand more about how research findings relate to their own lives and can offer more insights: ‘To collect information, yes, but also aimed at the possibility of creating a path for children. Let's say that the push is also to be able to think in some way to help them’ and ‘Everything that is research interests us… to give the best to our daughter, if there is some aspect that can be enhanced so that she can better fit in at school, she can become independent sooner or better…’.

#### 3.1.4 Opportunities to Receive Support

The theme ‘Opportunities to receive support’ was discussed in all nine focus groups and accounted for 10.69% of the discussions. Twenty‐seven participants (15 Italians and 12 Americans) mentioned the advantages of receiving guidance from specialists, both in person and online. They saw this support as an added value—not only for better understanding their child, but also as an opportunity to access professional expertise: ‘having a guide… that gives you that calm, tranquillity to reorganise future work is really a lifesaver’. They see research as a valuable source, especially in moments of uncertainty, helping them when they feel lost in addressing the demands of parenting a child with special needs and reinforcing their ongoing efforts to provide the best possible care: ‘I would have been very pleased to participate in research in this period […] a little bit maybe for the fact that I don't feel able to revolutionise my life, my knowledge, my interests to manage a child with special needs, so having my back covered by someone who has a higher, less involved, more objective look on what is the path of my son, would be a comfort to me’ and ‘…help and a guideline to show you the way a little bit when you're really lost and you don't know what to do is absolutely essential’.

#### 3.1.5 To Support the DS Community

Mentioned by 29 out of 33 participants (16 Italians and 13 Americans), the theme ‘To support the DS community’ emerged as a motivator across focus groups (9.10%). Parents described a sense of purpose in contributing to research projects, viewing their participation as a way to support the collection of unique data to which only they have access, and to share meaningful experiences: ‘participating in projects can be useful because a person who lives with Down syndrome firsthand can give information that can be useful’ and ‘I am pleased, even with an absolutely irrelevant contribution, to be able to be part of it, because I believe that sharing even only experiences is a fundamental starting point’. Another parent emphasised the importance of the cause of helping future families: ‘I like to think that my son can help other children in the future that are born with Down syndrome, at little…Little to no cost to him, just kind of effort from my part and his part of course… I believe that's a very important cause, I guess…Um, yeah, just for that's mine to help with future … Families … and kids’. As a consequence of the value they placed on shared information and experiences, many parents expressed a deep sense of gratitude for the support and knowledge provided by those who navigated the journey of raising a child with DS before them. This sense of having benefited from others fostered a desire to give back through research participation, in order to support future families of DS community: ‘I feel like we've benefited so much from the people that have kind of walked this path before us that it's a way for us to kind of give back to the Down syndrome community’ and ‘I want to help other kids with Down syndrome and their families’. Additionally, the feeling of being part of a broader change and actively contributing to it increases motivation, as it fosters a sense of responsibility to support other families facing similar challenges. One participant shared, ‘I want to make it easier for parents that are incoming um, because it is a big life change, so. My duty,’ whilst another said, ‘we participate in research because I think we can give our contribution both for ourselves and for the future… apart from her, there is a whole other group of children, who can benefit from this thing’.

#### 3.1.6 To Create a More Inclusive Society

The theme ‘To create a more inclusive society’ was the least mentioned across focus groups (3.70%), with 21 out of 33 participants (11 Italians and 10 Americans) referring to it at least once. Although it was the least frequently mentioned theme, this topic highlighted a significant motivation for some parents: the aspiration to contribute to a more inclusive society, both for their children and for future generations. For these parents, research is seen as a way to actively promote ‘… understanding and learning and building our knowledge base of how to, um, integrate people with disabilities into the community… it gets us better at that’, and to foster ‘the concept of integrating people with differences—disabilities—into general culture’. As one parent noted, ‘Everybody's fighting for—you know—advocating for – … you know full inclusion’. Many parents emphasised the ongoing efforts to promote inclusion and equal opportunities for individuals with Down syndrome, recognising that participation in research can be a powerful form of advocacy, driving systemic change and encouraging greater societal acceptance: ‘I like to participate in those things to help continue that progression of people with Down syndrome, … being accepted and being normalised into societies’.

### 3.2 Comparison Between Italian and American Focus Group Themes

**FIGURE 2:**
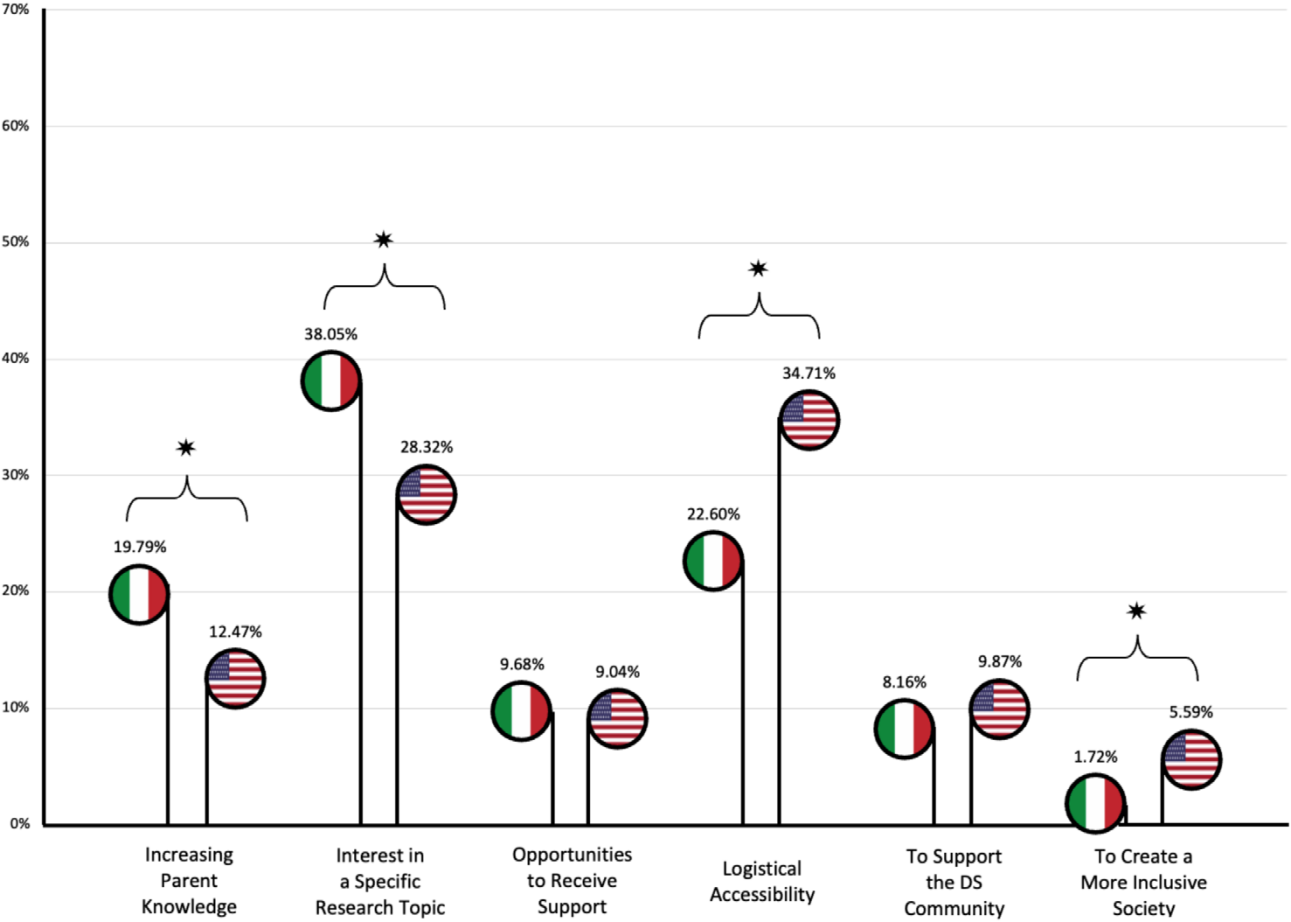
Comparison analysis between Italian and American focus group. *Note:* A ‘*’ has been placed above comparisons with significant differences.

The second quantitative analysis examined differences between American and Italian participants. Table 4 presents the frequencies of each theme discussed in the Italian and American focus groups. Figure 2 represents them graphically.

**TABLE 4:** Summary of the comparison between Italian and American frequencies.

	Italian parents	American parents
Word frequencies (%) *n*	180	141
Interest in a specific research topic	38.63%	29.44%
Logistical accessibility	105	152
	22.53%	31.73%
Increasing parent knowledge	88	57
	18.88%	11.90%
Opportunities to receive support	46	55
	9.87%	11.48%
To support the DS community	38	48
	8.15%	10.02%
To create a more inclusive society	9	26
	1.93%	5.43%
Number of parents that mention a theme (number of parents/total) *n* Interest in a specific research topic	19 out of 19	14 out of 14
Logistical accessibility	18 out of 19	14 out of 14
Increasing parent knowledge	18 out of 19	13 out of 14
Opportunities to receive support	15 out of 19	12 out of 14
To support the DS community	16 out of 19	13 out of 14
To create a more inclusive society	11 out of 19	10 out of 14

*Note:* Word frequencies were calculated by taking the mean between the frequencies in Table 3 for each theme in each country.

Overall, the most frequently discussed theme amongst Italian participants was ‘Interest in a specific research topic,’ with all 19 participants mentioning it, accounting for 38.63% of the coded quotations from Italian discussions. That was followed by ‘Logistical accessibility’ as the second most mentioned theme, cited by 18 out of 19 Italian participants, accounting for 22.53% of the quotations. Conversely, amongst American participants, ‘Logistical accessibility’ was the most frequently discussed theme, mentioned by all 14 respondents and comprising 31.73% of the quotations. Their second most frequently mentioned theme was ‘Interest in a specific research topic’, cited by every American participant, making up 29.44% of the quotations.

The same order of frequency between Italian and American participants was observed across the remaining four thematics. ‘Increasing parent knowledge’ ranked third, cited by 18 out of 19 Italian parents (18.88% of the quotations) and 13 out of 14 American parents (11.90% of the quotations). It was followed by ‘Opportunities to receive support’, mentioned by 15 out of 19 Italian participants (9.87% of the quotations), and 12 out of 14 American participants (11.48% of the quotations), which was the fourth most discussed thematic in both groups. ‘To support the DS Community’ was the fifth most mentioned theme, cited by 16 out of 19 Italian parents (8.15% of the quotations), and 13 out of 14 American parents (10.02% of quotations). Lastly, ‘To create a more inclusive society’ was the sixth most frequently cited theme, mentioned by 11 out of 19 Italian participants (1.93% of the quotations), and 10 out of 14 American participants (5.43% of the quotations).

Significant differences were observed between Italian and American participants in the distribution of quotation frequencies across the six themes, *χ* ^2^(2, 33) = 12.28, *p* < 0.0001. In particular, significant differences emerged with respect to the themes ‘Increasing parent knowledge’ (*χ* ^2^(1, *n* = 1055) = 9.29, *p* = 0.01) and ‘Interest in a specific research topic’ (*χ* ^2^(1, *n* = 1055) = 7.45, *p* = 0.024), which were more frequently cited by Italian participants. On the other hand, American participants more frequently discussed ‘Logistical accessibility’ (*χ* ^2^(1, *n* = 1055) = 10.75, *p* = 0.005) and ‘To create a more inclusive society’ (*χ* ^2^(1, *n* = 1055) = 8.28, *p* = 0.016) than Italian participants.

## 4 Discussion

The aim of this study was to identify parent motivations for participating in research on DS. The approach taken aligns with a CBPR framework, as applied to neurogenetic syndrome research (Riggs et al. 2020). CBPR calls for stronger partnerships between researchers and community members in the formulation of research agendas and study designs.

A critical first step in a CBPR partnership is to understand what brings community members to the research setting and why families choose to participate. In this study, a cross‐national sample of parents participated in focus group interviews, and their responses were transcribed and analysed to identify themes related to participant motivation. Nine focus group interviews were conducted with 33 participants (19 Italians and 14 Americans). The thematic analysis identified six main motivational themes: ‘Interest in a specific research topic’, ‘Logistical accessibility’, ‘Increasing parent knowledge’, ‘Opportunities to receive support’, ‘To support the DS community’, and ‘To create a more inclusive society’. Amongst these, the two most frequently mentioned were ‘Interest in a specific research topic’ and ‘Logistical accessibility’. These findings confirm earlier evidence that parents' willingness to engage in studies is shaped by the perceived usefulness and personal relevance of the study (Reines et al. 2017; White et al. 2022), including its alignment with the child's age and developmental stage (Amirav et al. 2017), but also by practical and structural constraints on participation. Consistent with prior work (Reines et al. 2017; White et al. 2022; Bardhan et al. 2024), participants identified travel distance, time commitment, and scheduling difficulties as major barriers. Notably, focus groups with a higher proportion of working parents raised logistical concerns more frequently (e.g., 46.34%, American focus group 9), suggesting that availability and accessibility play a key role in shaping participation.

‘Interest in a specific research topic’ and ‘Logistical accessibility’ also reflect key CBPR principles of ensuring that research priorities are responsive to community interests and tailored to participants' practical needs (Israel et al. 1998; Riggs et al. 2020). Indeed, interview responses indicated that home‐based interventions—offering greater accessibility and flexibility—can significantly enhance parental interest in participation. Some families may require additional support or accommodations to facilitate their participation in research. Collaborating with families to identify convenient times (e.g., evenings, weekends, school holidays) and suitable locations (e.g., home visits, nearby study sites) could significantly enhance the recruitment of more representative samples in research on DS.

For many parents, research was also perceived as a source of emotional and practical guidance, offering reassurance and direction during challenging times (‘Opportunities to receive support’). This finding echoes previous evidence showing that parents value the sense of support and connection with experts that research can provide (Williams et al. 2018). Together with parents' interest in expanding their knowledge, particularly about DS (‘Increasing parent knowledge’), these two motivations align with core CBPR principles that emphasise collaboration and the co‐creation of knowledge (Israel et al. 1998; Riggs et al. 2020). At the same time, parents were aware of their role in contributing valuable information to the research process, a perspective that reflects another CBPR principle: the importance of collaborative relationships that address the community's expressed needs (Riggs et al. 2020).

In line with prior literature (Burstein et al. 2014; Menon et al. (2025); Reines et al. 2017; White et al. 2022; Williams et al. 2018), parents also described altruistic motivations, such as the desire to help other families, to ‘give back’ to the DS community, or to advance future research (‘To support the DS community’). Moreover, they expressed an aspiration to contribute to broader social change towards greater inclusion (‘To create a more inclusive society’). These accounts suggest that altruism remains an important driver of parental motivation across different research contexts, as it reflects parents' willingness to support future families and foster collective progress rather than solely address individual needs. Additionally, these themes reflect CBPR principles emphasising shared impact and the alignment of research goals with both community‐identified priorities and broader social values (Israel et al. 1998, 2010; Riggs et al. 2020).

Compared with prior studies predominantly conducted in medical contexts in the US, our findings from Italian and US families revealed many similarities regarding the motivations that enhance research participation, such as the personal relevance of the study, perceived benefits for children and parents, contribution to the DS community, and the logistical accessibility of the research. At the same time, some differences also emerged, particularly motivations related to the opportunity to learn new things about their children or to promote greater inclusion of individuals with DS in society, as well as the commitment required to continue attending the research sessions and the emotional effort demanded of the child during participation. These aspects appear to be more closely associated with themes and issues typically explored in psychological research and are somehow different in studies focusing more on genomic research or pharmacological trials.

The alignment between parents' motivations for participating in research and CBPR principles is particularly important, as it reinforces a shared vision of research as a collaborative and responsive process. When the community is actively involved, participants are more motivated because they can express their needs and help shape the research agenda, resulting in greater legitimacy and acceptance of the process. At the same time, researchers gain access to timely, direct data that reflect the lived realities and practical priorities of the population under study. This leads to findings that are more valid and grounded, more applicable to interventions, and more representative, thanks to higher participation and stronger adherence.

### 4.1 Similarities Between Countries

The analysis of the focus groups also aimed to determine whether the themes mentioned by parents generalised across countries or reflected context‐related factors influencing research participation. Recognising similarities between two distinct cultures might assist researchers in determining which elements to prioritise when designing a study. Notably, the two most frequently mentioned themes—'Interest in a specific research topic’ and ‘Logistical accessibility’—were the same in both Italy and the US, although cited in reverse order, whereas the remaining themes followed the same frequency ranking across both. These findings suggest a fundamental similarity between the two groups. Despite contextual differences in geography, daily routines, and work structures, the motivational themes that emerged when participants were asked to describe the factors they consider before agreeing to take part in a study appeared largely consistent across the two countries. In particular, ‘Interest in a specific research topic’ was the most frequently mentioned theme amongst both Italian and American participants. All respondents referred to this theme in some form, indicating that research goals play a central role in shaping motivation. These findings build on previous literature reporting similar results (Hodapp et al. 2001) and, in line with CBPR principles, highlight key factors that researchers may consider when designing studies – factors which, when taken into account, can help foster greater participant engagement.

‘To support the DS community’ was one of the least frequently mentioned motivations by both American and Italian participants. The focus group with the highest citation rate is number seven, which also has the lowest average age of the members. This could suggest a generational issue: whereas younger participants believe they are more responsible for promoting social change, older participants believe they have already contributed.

### 4.2 Differences Between Countries

Understanding how two cultures differ can help researchers identify key cultural factors to consider, especially when conducting parallel studies across countries or adapting research developed in different cultural contexts. Italian participants appeared to be more motivated than their American counterparts by the desire to increase their knowledge in a specific area (‘Increasing parent knowledge’). This may be partly due to the fact that three Italian participants had backgrounds in other academic fields, which might have influenced the nature of the discussions. This observation suggests that participants' perspectives should be interpreted carefully, as their personal and professional experiences may sometimes shape their views in ways that differ from those of the broader community.

Moreover, the results showed that between the two most frequently mentioned themes, Italian participants had a significantly greater preference for ‘Interest in a specific research topic’ compared to American participants. The opposite pattern was observed for the theme ‘Logistical accessibility’. Possible explanations may relate to the fact that in US geographic distance from health care locations causes a delay or lack of access to diagnosis and services. As a result, being close to research locations becomes critical. This issue is less common in Italy, where the general proximity between rural and urban areas allows parents to focus more on their topic preferences and personal needs, rather than on potential logistical barriers.

## 5 Implications

This study aimed to investigate the motivations that lead parents to participate in research. Identifying the factors that drive parental decision‐making can help researchers better tailor study design to family interests and values. Balancing these priorities with the scientific objectives of the study is essential for creating inclusive and effective research designs. When research goals are informed by parent priorities, they can become more responsive to community needs, which in turn may increase adherence, engagement, and the relevance of findings.

## 6 Limitations and Future Directions

Given the qualitative and interpretive nature of this study, it is important to consider the potential influence of the researchers' sociocultural positions on the research process. Their professional and cultural perspectives may have shaped the facilitation of focus groups as well as the interpretation of participants' responses. Although efforts were made to minimise bias in the present study—such as employing bilingual coders, cross‐national collaboration, and iterative discussions between research teams—our positionalities may still have limited the range of interpretations considered.

Moreover, our study team's prior commitment to CBPR may have influenced both the framing of the focus group questions and the interpretation of the findings, and we report it here in the interest of transparency. The focus groups were designed to explore parents' motivations for participating in research and to provide input for the development of a novel cross‐national intervention. Accordingly, in analysing the results, we explicitly linked participants' responses to CBPR principles and to potential modes of involvement in an intervention.

Another limitation concerns the group setting itself. Social desirability bias may have played a role, as parents in a focus group context might have emphasised responses that appeared more favourable or socially acceptable to others in the group, as most of them were not previously acquainted with one another. Additionally, because some participants were directly contacted via email by the research team or through local advocacy organisations, the sample may have included families who were already motivated or particularly willing to engage in research, which may have influenced their responses.

Discrepancies in group size between countries may have affected social dynamics. Specifically, due to logistical factors, the American focus groups were conducted with a maximum number of participants of three, whereas Italian focus groups were composed of six to seven participants. Furthermore, because four out of six US focus groups consisted of only two parents, the conversation may have been less influenced by interpersonal variability than in focus groups with six or seven participants. Future research may balance this variable to examine if the frequency of word themes is still unchanged or modified. These findings were obtained by recruiting mostly parents of preschool‐aged children with DS. To generate more representative data, it may be necessary to either establish a baseline with parents of children without disabilities or to recruit participants of school‐aged children with DS. Future research should also include samples of parents of children with other neurogenetic conditions associated with IDD. Furthermore, both Italy and the US are individualistic cultures; it would be useful to conduct the same study between an individual and a collectivistic culture to observe if and by how much the themes more oriented to others would change.

## 7 Conclusions

Findings from this study can help bridge the gap between community members and researchers by comparing the key factors that most strongly motivate parents to participate in research across different cultural contexts (American and Italian). Quantitative focus group analysis was used as a methodological tool. Word frequencies were collected to generate comparable data whilst also providing a rich and informative contextual framework in which to locate them. The analysis of theme frequencies revealed that ‘Interest in a specific research topic’ was the most prominent motivator amongst Italian participants, whereas ‘Logistical accessibility’ emerged as the most salient theme for American participants. Some notable cultural differences were identified, suggesting that parental priorities vary between the two countries and that the six themes did not carry the same level of importance across contexts. Whilst the relative importance of specific themes varied between the two countries, the overall ranking of themes by frequency was largely similar across them, suggesting a shared underlying structure in parental motivations.

These findings underscore the importance of tailoring research approaches to community‐specific priorities, in line with the principles of CBPR. Accordingly, the current study contributes to the growing body of work applying CBPR frameworks in research on neurogenetic conditions (Riggs et al. 2020). Finally, the information gathered in this study is intended to contribute to a growing field of research that prioritises families' engagement and responsiveness to the community's needs. Scientific research should recognise the input provided by the target community not only as a crucial starting point, but also as valuable feedback to guide future directions.

## Author Contributions

Conception and design: Benedetta Ceci, Madison M. Walsh, Sara Colaianni, Francesca Pulina, Silvia Lanfranchi, and Deborah J. Fidler. Data analysis and interpretation: Benedetta Ceci, Madison M. Walsh, Bethany A. Gray, Sara Colaianni, Erika Lupati, Alessandra Tomè, Margherita Pietrobon, Silvia Lanfranchi, and Deborah J. Fidler. Manuscript drafting: Benedetta Ceci, Sara Colaianni, Madison M. Walsh, Silvia Lanfranchi, and Deborah J. Fidler. Manuscript review and editing: Benedetta Ceci, Madison M. Walsh, Sara Colaianni, Miranda E. Pinks, Sara Onnivello, Kaylyn Van Deusen, Francesca Pulina, Chiara Marcolin, Bethany A. Gray, Elisa Rossi, Erika Lupati, Margherita Pietrobon, Alessandra Tomè, Deborah J. Fidler, Silvia Lanfranchi.

## Funding

This work was supported by Fondation Jérôme Lejeune (Project #2061) and the National Institutes of Health (Award #R61 HD115161).

## Ethics Statement

The study protocol and procedures were approved by the Institutional Review Board (IRB) at Colorado State University and the University of Padua Ethics Committee. Participants gave informed consent prior to the initiation of any study activities. The study was performed in accordance with the Declaration of Helsinki.

## Conflicts of Interest

The authors declare no conflicts of interest.

## Data Availability

The data that support the findings of this study are available from the corresponding author upon reasonable request.
